# Harnessing the neuroplastic potential of the human brain & the future of cognitive rehabilitation

**DOI:** 10.3389/fnhum.2014.00218

**Published:** 2014-04-11

**Authors:** Jyoti Mishra, Adam Gazzaley

**Affiliations:** Department of Neurology, Physiology and Psychiatry, University of CaliforniaSan Francisco, San Francisco, CA, USA

**Keywords:** cognitive training, neurotherapeutics, cognitive control, neuroplasticity, closed loop

Neuroplasticity is the remarkable ability of the brain that allows us to learn and adapt to our environment. Many studies have now shown that plasticity is retained throughout the lifespan from infancy to very old age (Merzenich et al., 1991; Merzenich and DeCharms, 1996; Greenwood and Parasuraman, 2010; May, 2011; Bavelier et al., 2012). Enriching life experiences, including literacy, prolonged engagement in the arts, sciences and music, meditation and aerobic physical activities have all been shown to engender positive neuroplasticity that boosts cognitive function and/or prevents cognitive loss (Vance et al., 2010; Hayes et al., 2013; Matta Mello Portugal et al., 2013; Newberg et al., 2013; Zatorre, 2013). Unfortunately, just as enriching experiences generate positive plasticity, negative plasticity ensues in impoverished settings. For instance, many studies now show that low socio-economic, resource-poor environments, which are associated with stress, violence and abuse within families and communities, have detrimental effects on cognition and neural function (D'Angiulli et al., 2012; McEwen and Morrison, 2013). As cognitive neuroscientists we observe both positive and negative aspects of plasticity in neural systems, in functional changes of neural activations, neural oscillations and strength of connectivity between brain regions, in structural changes in gray matter volume and white matter integrity, and importantly in the relationship between such neuroplastic changes and concomitant cognitive/behavioral changes. As we come to understand various facets of plasticity, it drives further the quest to develop new activities/interventions that engender maximal positive plasticity in selectively targeted neural systems; we envision such activities will in turn generate “far transfer of benefit” to generalized cognition and thereby improve the human condition.

In today's modern technological and internet-connected era, individuals are increasingly engaging with cognitive training software to improve cognitive function. In fact over the past 10–15 years, several companies have become established proponents and marketers of such software, transforming it into a multi-million dollar industry with exponential projected future growth. The fact that this technology is easily accessible over the internet to the home-setting, and at low-cost, has facilitated it's mass adoption. Scientifically, however, not all “brain training” is made equal. All too often, basic cognitive neuroscience experimental paradigms are embedded in commercial “brain training” approaches with add-on visual graphic skins that attempt to maximize user-engagement; a process known as gamification. Although these experimental paradigms had been originally developed to understand cognition, that does not mean that they are also the best tools to engender positive neuroplasticity. It is no surprise then that some scientific investigations have uncovered that generic brain training approaches yield no positive cognitive outcomes (Owen et al., 2010). However, a blanket statement that all cognitive training is ineffective is also unfair. In recent years, development and evaluation of cognitive training approaches in many labs, including our own, has revealed evidence for positive neuroplasticity, as well as for transfer of benefit to untrained cognitive abilities (Tallal et al., 1996; Temple et al., 2003; Stevens et al., 2008; Smith et al., 2009; Ball et al., 2010; Berry et al., 2010; Anderson et al., 2013; Anguera et al., 2013; Mishra et al., 2013; Wolinsky et al., 2013). Furthermore, in two of our training studies we find neurobehavioral correlations that relate on-task neuroplasticity to broader improvements in untrained aspects of cognition. Other researchers have also reported positive findings and transfer of training effects to untrained cognitive abilities in the context of custom-designed working memory exercises (Klingberg, 2010; Rutledge et al., 2012), task-switching training (Karbach and Kray, 2009), as well as for a specific genre of commercially available games, i.e., action video games (Bavelier et al., 2012) (although it is difficult to make strong recommendations about many off-the-shelf games given concerns over violent content). From these studies we are coming to understand some of the design principles that may govern the development of effective neuroplasticity-targeted training, as well as the scientific evaluation methods that can be used to provide convincing proof of the efficacy of the training intervention. Here, we summarize some of these principles that have emerged from two of our published training studies that now inform the development and evaluation of our next generation of training tools.

In our first training study in older adults, we simply trained visual perceptual discrimination of Gabor patches that had built-in directed motion animation (Berry et al., 2010). Ten hours of training improved on-task perception relative to performance changes in a non-training (no-contact) control group. Interestingly, the training also benefitted delayed-recognition working memory of an untrained motion direction task. Not only was working memory performance improved, electroencephalography (EEG) neural recordings showed that training evoked more efficient sensory encoding of the stimuli, which correlated with the working memory performance gains. This finding that 10 h of simple perceptual training engendered transfer of benefits to working memory aligns with recent understanding that perceptual training improves signal to noise contrast, which then leads to refined encoding at multiple neural scales and hence, at least some degree of generalized cognitive benefits (Vinogradov et al., 2012).

We are now gaining an appreciation that the observed gains in our perceptual training study, and in similar studies performed by other labs, some of which have shown long-lasting cognitive benefits (Willis et al., 2006; Rebok et al., 2014), may be mediated by two fundamental design elements that drive neuroplasticity. 1) Training incorporated continuous performance feedback at multiple levels of game play providing repeated cycles of reward to the user 2) Training was adaptive to the trainee's in-the-moment game performance; i.e., adaptivity was incorporated using psychophysical staircase functions that enhance training challenge in response to accurate performance and reduce it for inaccurate performance. The up-down step ratio in such staircases is often chosen to maintain overall task challenge at 75–85%, at which point the user is optimally engaged but not frustrated. Thus, continuous performance feedback rewards and adaptive task challenge uniquely personalize the training to the cognitive capacity of each individual, and allows abilities to improve over time. Overall we have found these features to be critically important in generating positive neuroplasticity and cognitive benefit. Note, it is important to realize that casual game software is often not designed to provide the optimal dose of repetitive rewards nor incorporate adaptive progressions specifically targeted to the cognitive domains that may be deficient in a given population cohort. These factors, along with the heterogeneity of tested populations, very small training doses on multiple cognitive exercises, and the use of assessment measures that are insensitive to detect training related benefits in the tested population, all may contribute to a failure to observe positive impact (e.g., Owen et al., 2010).

While reward cycles and adaptive progressions are key components of software design, it is equally important to tailor these game mechanics toward improving specific deficits observed in a population cohort. For instance, Anguera et al. (2013) showed that deficient cognitive control abilities, such as working memory and sustained attention, in healthy older adults can be enhanced by specifically training on a multitasking performance-adaptive and rewarding video game, “*Neuroracer.”* “*Neuroracer*” implements visual discrimination training in a go-no-go task for colored shape targets, with the added demand of simultaneously driving on a virtual road. “*Neuroracer*” evidenced extensive gains such that healthy older adults who multi-tasked 175% worse than younger adults on a first assessment, achieved significant post-training performance levels on the game itself that surpassed those of young adults by +44%. Importantly, training on “*Neuroracer*” transferred to untrained measures of sustained attention and working memory in the setting of interference, with EEG-based neural recordings showing that plasticity of midline frontal theta (mf theta) neural oscillations may be a mediator of these cognitive improvements.

While we have tested some aspects of sound game design, as described, other aspects of high-level video games may contribute to their success and we look ahead to assessing these empirically. For example, immersion, fun, real-world features, continuous performance, 3D environments, virtual reality, high-levels of art, story, and music facilitate sustained performance and better compliance, and also deeper engagement that we suspect maximally harnesses plasticity. Evaluation of the influence of these features on training effectiveness requires careful scientific study design. For this, the “*Neuroracer*” study adopted a rigorous three-armed randomized controlled design. In addition to the multitasking training group, the study included an active single-task training control, as well as a no-contact control group. The single-task training control performed the exact same tasks as the training group of visual discrimination and driving, except that task engagement was not concurrent. This active control directly tested our hypothesis that only training in a setting that stresses cognitive control via a high interference environment would show significant cognitive gains. Outcomes of the “Neuroracer” multitasking training were not achieved in the active control group or in the no-contact group, the latter being critical for assessing practice effects due to repeated evaluations. Thus, the “*Neuroracer*” study highlighted that rigorous scientific evaluation of a cognitive training approach requires appropriate control groups, and often more than one control group, especially if we want to understand the underlying mechanisms of training effectiveness. Indeed longitudinal data collection is arduous, but without randomized, controlled and single/double blinded enrollments, we cannot convince ourselves of the significance of the results of new interventions. This is especially appropriate for healthy populations, while single-arm feasibility trials do remain informative as a first pass in cognitively impaired populations. In addition, we should also implement expectation bias measures for all participants, which confirm that all study groups anticipate the same level of influence of their assigned intervention on the outcome measures, thus assuring appropriate placebo control (Boot et al., 2013). Finally, adequately powered large sample size studies and investigations that measure sustainability of the cognitive gains and underlying neuroplasticity in yearly follow-ups are rare and need to be performed more often to address the long-term efficacy of cognitive interventions. Such rigor is convention in pharmaceutical clinical trials, and its adoption for video game testing, along with safety evaluations that detect potential side effects such as game-addiction, would promote a path toward FDA approvals and medical prescription of such technologies.

Equipped with our growing understanding of how to design cognitive training approaches to target plasticity in specific neural circuits, we are now embarking on the development of the next generation training technologies. We envision these advances to include combining behavior-digital closed loops that link behavioral performance metrics to adaptive modulations of a training task on a digital platform, with neuro-digital closed loops that link neural performance measures to adaptive game mechanics. For example, the “*Neuroracer*” training study discovered that neuroplasticity of midline frontal theta (enhanced mf theta post-training) is a key neural factor that correlates with transferred cognitive gains. In order to test whether mf theta plasticity is truly causal in enabling improved cognition, we are now developing neuro-digital closed loops that directly target mf theta activity. More specifically, technological development is being directed at real-time EEG-based recordings that occur simultaneous with the cognitive task training (Delorme et al., 2011; Makeig et al., 2012; Kothe and Makeig, 2013). The goal is for these measurements to be event-locked to task stimuli, account for ocular and muscle-related artifacts, and use source localization algorithms (Mullen et al., 2013) so that they can be directly integrated in the game environment to guide reward feedback to the user and adaptivity of task challenge in real-time. We hypothesize that using neural performance as the driver for task-adaptivity will generate more rapid, efficient and specific circuit plasticity than is currently obtained using behavior-adaptive cognitive training approaches. This hypothesis is borne out of data, which shows that single-trial behavioral performance is predicted by neural measures such as mf-theta oscillations preceding the behavior. Thus the neuro-digital closed loop offers the potential to selectively train and refine the bottleneck neural processes that govern the final behavioral outcome. Importantly, by directly embedding task-related neural activity in a closed loop, this approach can provide missing causal evidence between neuroplasticity and cognitive benefits. This line of investigation is especially promising in the light of accumulating scientific evidence of the value of conventional neurofeedback approaches (Gruzelier, 2013; Wang and Hsieh, 2013; Arns et al., 2014), which also creates a neuro-digital closed loop, albeit driven by ongoing scalp EEG oscillations as opposed to task-related neural processes as we envision.

We are aware that unlike traditional cognitive training, a neuro-digital closed loop approach is not feasible as a mainstay in the home setting at present. Yet, with rapid developments of mobile EEG technology (Stopczynski et al., 2013), as well as advances in the real-time computational power available on consumer devices such as laptops and tablets, we expect that deployment in the home environment will be a reality within a few years. Neuro-digital closed loops are also an exciting way to achieve personalized therapeutics, as each feedback loop is customized to the individual user's neural capacities in the moment. While here we have provided a simplistic example of a closed loop tied to task-related mf theta activation, one can conceive of more sophisticated neural targets, including frontal-posterior effective connectivity based on task interaction dynamics. Further advances in this field are expected as neuroscientists collaborate with neural engineers, who have predominantly focused related efforts on neuroprosthetic development (Borton et al., 2013). Neuro-engineers have designed efficient closed loop decoding algorithms for brain-machine interfaces in animal model systems, and these techniques are now ripe for adoption in humans (Carmena, 2013). Finally, especially beneficial for clinical populations that exhibit weakened neural responsivity, another intriguing step will be the integration of neuro-digital closed loop systems with transcranial electrical current stimulation or even deep brain stimulation technologies (Coffman et al., 2013), which may provide a needed plasticity boost to impaired brain regions.

To achieve the goals of our field and fully harness the potential of neuroplasticity for cognitive benefit, we look forward to continued technological development, such as neuro-digital closed loops, and their integration with emerging design principles of cognitive training games. These technologies validated using randomized, controlled scientific evaluation methodologies will generate new understanding of how to translate cognitive neuroscience discoveries into new educational tools for healthy populations and mental healthcare interventions for neuropsychiatric populations in need of cognitive remediation.

## Conflict of interest statement

Jyoti Mishra is a part-time scientist at the Brain Plasticity Institute, PositScience, a company that develops cognitive training software. Adam Gazzaley is co-founder and chief science advisor of Akili Interactive Labs, a company that develops cognitive training software. Jyoti Mishra and Adam Gazzaley have a patent pending for “Methods of Suppressing Irrelevant Stimuli.” Adam Gazzaley has a patent pending for a game-based cognitive training intervention: “Enhancing cognition in the presence of distraction and/or interruption.”
